# Genetic, transcriptional, and regulatory landscape of monolignol biosynthesis pathway in *Miscanthus* × *giganteus*

**DOI:** 10.1186/s13068-020-01819-4

**Published:** 2020-10-27

**Authors:** Xiaofei Zeng, Jiajing Sheng, Fenglin Zhu, Tianzi Wei, Lingling Zhao, Xiaohu Hu, Xingfei Zheng, Fasong Zhou, Zhongli Hu, Ying Diao, Surong Jin

**Affiliations:** 1 School of Biology and Pharmaceutical Engineering, Wuhan Polytechnic University, Wuhan, 430023 People’s Republic of China; 2 School of Medicine, Southern University of Science and Technology, Shenzhen, 518055 People’s Republic of China; 3 School of Life Sciences, Nantong University, Nantong, 226019 People’s Republic of China; 4 State Key Laboratory of Hybrid Rice, College of Life Sciences, Hubei Lotus Engineering Center, Wuhan University, Wuhan, 430072 People’s Republic of China; 5 School of Chemistry, Chemical Engineering and Life Sciences, Wuhan University of Technology, Wuhan, 430070 People’s Republic of China

**Keywords:** *Miscanthus* × *giganteus*, Bioethanol, Lignin, Monolignol biosynthesis pathway, Monolignol biosynthetic genes, Transcription factors, Regulatory mechanism, Transcriptome analysis, Genetic engineering

## Abstract

**Background:**

*Miscanthus* × *giganteus* is widely recognized as a promising lignocellulosic biomass crop due to its advantages of high biomass production, low environmental impacts, and the potential to be cultivated on marginal land. However, the high costs of bioethanol production still limit the current commercialization of lignocellulosic bioethanol. The lignin in the cell wall and its by-products released in the pretreatment step is the main component inhibiting the enzymatic reactions in the saccharification and fermentation processes. Hence, genetic modification of the genes involved in lignin biosynthesis could be a feasible strategy to overcome this barrier by manipulating the lignin content and composition of *M*. × *giganteus*. For this purpose, the essential knowledge of these genes and understanding the underlying regulatory mechanisms in *M*. × *giganteus* is required.

**Results:**

In this study, *MgPAL1*, *MgPAL5*, *Mg4CL1*, *Mg4CL3*, *MgHCT1*, *MgHCT2*, *MgC3′H1*, *MgCCoAOMT1*, *MgCCoAOMT3*, *MgCCR1*, *MgCCR2*, *MgF5H*, *MgCOMT*, and *MgCAD* were identified as the major monolignol biosynthetic genes in *M*. × *giganteus* based on genetic and transcriptional evidence. Among them, 12 genes were cloned and sequenced. By combining transcription factor binding site prediction and expression correlation analysis, MYB46, MYB61, MYB63, WRKY24, WRKY35, WRKY12, ERF021, ERF058, and ERF017 were inferred to regulate the expression of these genes directly. On the basis of these results, an integrated model was summarized to depict the monolignol biosynthesis pathway and the underlying regulatory mechanism in *M*. × *giganteus*.

**Conclusions:**

This study provides a list of potential gene targets for genetic improvement of lignocellulosic biomass quality of *M*. × *giganteus*, and reveals the genetic, transcriptional, and regulatory landscape of the monolignol biosynthesis pathway in *M*. × *giganteus*.
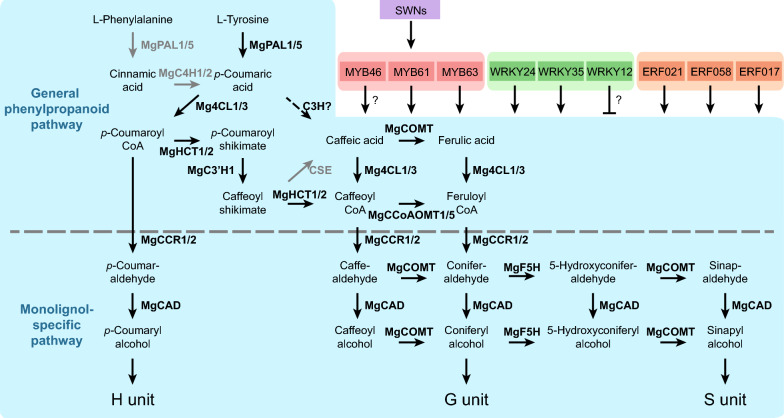

## Background

*Miscanthus* × *giganteus* is a triploid perennial rhizomatous C_4_ grass that originated from the natural hybridization between diploid *Miscanthus sinensis* and tetraploid *Miscanthus sacchariflorus* [1]. Owing to its outstanding features, such as high biomass production [2], low environmental impacts [3], and the potential to be cultivated on marginal land [4], *M*. × *giganteus* is widely recognized as a promising lignocellulosic biomass crop for bioethanol production. However, the recalcitrant nature of lignocellulosic feedstocks leads to the high costs of pretreatment, saccharification, and fermentation processes, limiting the current commercialization of lignocellulosic bioethanol [5, 6].

Among the biopolymers in lignocellulosic biomass, the lignin enriched in the secondary cell wall is one of the main factors that account for recalcitrance [7]. Besides, its by-products released in the pretreatment step are the primary inhibitors of enzymatic reactions in the saccharification and fermentation processes [8, 9]. To overcome this barrier, researchers have focused on manipulating the lignin content and composition of the lignocellulosic feedstocks via genetic engineering approaches. In maize [10, 11] and switchgrass [12–14], the reduction of lignin content and optimization of lignin composition significantly promoted the saccharification efficiency and ethanol productivity. A similar correlation was observed in the natural *Miscanthus* accessions [15, 16]. Additionally, the sterile nature of triploid *M*. × *giganteus* enhances the environmental safety of genetic engineering. These results suggest the potential utilization of genetic manipulation of lignin biosynthesis in *M*. × *giganteus*. To this end, basic knowledge of the genes involved in lignin biosynthesis and how these genes are regulated in *M*. × *giganteus* is essential.

**Fig. 1:**
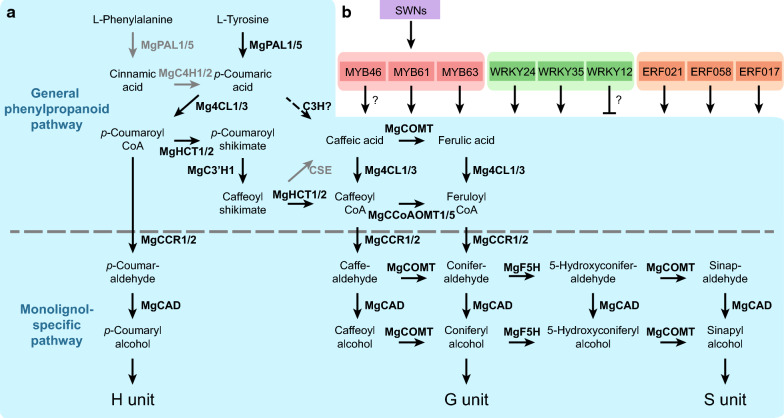
Integrated model of monolignol biosynthesis pathway and the underlying regulatory mechanism in *Miscanthus* × *giganteus*. **a** The proposed monolignol biosynthesis pathway in *M*. × *giganteus*. The enzymes beside the arrows are the major monolignol biosynthetic enzymes identified in this study. The gray arrows indicate the enzymatic reactions are not supported (CSE) or not the main route (from phenylalanine to *p*-coumaric acid). The dashed arrow means the reaction is not yet clear in *M*. × *giganteus* (C3H). **b** The transcription factors (TFs) that are predicted to participate in the monolignol biosynthesis in *M*. × *giganteus*. The lines with arrows and bars at the ends represent promoting and suppressing the expression of monolignol biosynthetic genes, respectively. The MYBs, WRKYs, and ERFs are likely to directly regulate the expression of monolignol biosynthetic genes, while the secondary wall NACs (SWNs) are supposed to function indirectly. For each family, the top three TFs are shown in the figure, and the rest are listed in Additional file 2: Table S9

The lignin in the cell wall is mainly composed of *p*-hydroxyphenyl (H), guaiacyl (G), and syringyl (S) units, which are polymerized from the corresponding monolignols, *p*-coumaryl, coniferyl, and sinapyl alcohols, respectively. In flowering plants, these monolignols are synthesized from the general phenylpropanoid pathway and the following monolignol-specific pathway, as shown in Fig. 1a [17]. Phenylalanine ammonia-lyase (PAL) and cinnamic acid 4-hydroxylase (C4H) are the first two enzymes in the general phenylpropanoid pathway catalyzing the synthesis of *p*-coumaric acid from phenylalanine. Recently, a bi-functional cytosolic ascorbate peroxidase (APX) was reported to function as 4-coumarate 3-hydroxylase (C3H) synthesizing caffeic acid through the 3-hydroxylation of *p*-coumaric acid [18]. The subsequent 3-*O*-methylation from caffeic acid to ferulic acid is catalyzed by caffeic acid/5-hydroxyconiferaldehyde 3/5-*O*-methyltransferase (COMT). Then, these hydroxycinnamates are converted to the corresponding CoA esters by 4-hydroxycinnamate: CoA ligase (4CL). A more generally accepted route for caffeoyl CoA synthesis is derived from *p*-coumaroyl CoA via the catalyzing of hydroxycinnamoyl CoA: shikimate hydroxycinnamoyl transferase (HCT) and coumaroyl shikimate 3′-hydroxylase (C3′H). The resulting caffeoyl CoA is 3-*O*-methylated by caffeoyl CoA 3-*O*-methyltransferase (CCoAOMT). In the monolignol-specific pathway, these CoA esters are converted to corresponding aldehydes and alcohols by cinnamoyl CoA reductase (CCR) and cinnamyl alcohol dehydrogenase (CAD), respectively. Besides, some enzymes in the monolignol biosynthesis pathway are involved in the flux from G to S monolignol at the levels of aldehydes and alcohols, including COMT and ferulic acid/coniferaldehyde 5-hydroxylase (F5H).

However, the monolignol biosynthesis pathway is variable across different species. In some monocots, PAL also exhibits the capability to catalyze the non-oxidative elimination of ammonia from tyrosine to *p*-coumaric acid directly, known as the tyrosine shortcut pathway [19–22]. Another example is caffeoyl shikimate esterase (CSE). It converts caffeoyl CoA to caffeic acid in some species, while its orthologs are absent in the genomes of *Brachypodium distachyon*, *Zea mays*, and *Sorghum bicolor* [23]. This diversity highlights the need to understand the monolignol biosynthesis pathway in every lignocellulosic crop.

Indeed, some studies have focused on the genetic and regulatory basis of the monolignol biosynthesis pathway in some bioenergy grasses [24–28]. However, in *Miscanthus* species, comprehensive research is still lacking, and no integrated model has been built yet. To fill this gap, we identified the major monolignol biosynthetic genes in *M*. × *giganteus* and predicted the probable transcription factors (TFs) that directly regulate these genes. Based on these results, an integrated model was summarized to depict the monolignol biosynthesis pathway and the underlying regulatory mechanism (Fig. 1a, b). This study provides a list of potential gene targets for genetic improvement of lignocellulosic biomass quality of *M*. × *giganteus*. Also, it reveals the genetic, transcriptional, and regulatory landscape of the monolignol biosynthesis pathway in *M*. × *giganteus*.

## Results

### Evolutionary history of monolignol biosynthetic genes in angiosperms

The cDNAs of 20 monolignol biosynthetic genes were cloned and sequenced from *M*. × *giganteus*. The basic information and GenBank accession number of each cDNA are listed in Table 1.

**Table 1:** Summary of the monolignol biosynthetic genes cloned from *Miscanthus* × *giganteus* in this work

Gene name	Total length (bp)	5′-UTR length (bp)	ORF length (bp)	3′-UTR length (bp)	Protein length (aa)	GenBank accession number
*MgPAL1*	2232	16	2115	101	704	KX084997
*MgPAL2*	2157	9	2145	3	714	KX084998
*MgPAL3*	2596	152	2160	284	719	KX084999
*MgPAL5*	2182	70	2112	0	703	KX085000
*MgC4H1*	1506	0	1506	0	501	KT933125
*MgC4H2*	1518	0	1518	0	505	KT933126
*Mg4CL1*	1933	3	1665	265	554	KX085001
*Mg4CL4*	1650	0	1647	3	548	KX085002
*MgHCT1*	1,722	74	1323	325	440	KT933127
*MgCCoAOMT1*	786	0	786	0	261	KX085003
*MgCCoAOMT3*	751	2	741	8	246	KX085004
*MgC3′H1*	1541	2	1539	0	512	KT933128
*MgC3′H2*	1542	0	1542	0	513	KT933129
*MgCCR1*	1460	146	1119	195	372	KP828433
*MgCCR2*	1437	161	1089	187	362	KP828436
*MgCCR3*	1093	0	1032	61	343	KT933131
*MgCCR4*	1035	0	1035	0	344	KT933132
*MgF5H*	1648	0	1590	58	529	KT933130
*MgCOMT*	1494	101	1089	304	362	KP717904
*MgCAD*	1506	159	1098	249	365	KP793695

**Fig. 2:**
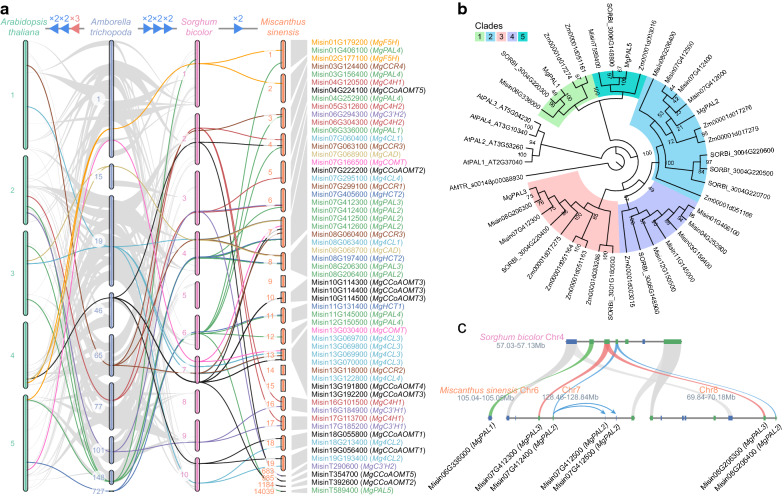
Phylogenetic and genome synteny analyses. **a** The macro-synteny plot to show the orthologous relationships of the monolignol biosynthetic genes across different species at the chromosome level. The genes in the parentheses are the corresponding orthologs in *Miscanthus* × *giganteus*. The blue and red triangles indicate whole-genome duplication and triplication events, respectively. **b** The phylogenetic tree of PALs. The numbers beside the nodes are ultrafast bootstrap values. The five clades were filled with different colors. The genes starting with “AMTR”, “At”, “SORBI”, “Zm”, “Misin” and “Mg” are from *Amborella trichopoda*, *Arabidopsis thaliana*, *Sorghum bicolor*, *Zea mays*, *Miscanthus sinensis*, and *M*. × *giganteus*, respectively. **c** The micro-synteny plot to illustrate the duplication history of *MgPAL2* and *MgPAL3*. The gray bars indicate the chromosomal regions. The blue and green rectangles represent the genes on the plus ( +) and minus (−) strands, respectively. The tandem duplication and whole-genome duplication events are shown as ribbons and arrows

The orthologous relationships of genes could provide evidence for inferring the gene evolutionary history and functions [29]. For this reason, we performed the genome synteny and phylogenetic analyses on the monolignol biosynthetic genes from the basal angiosperm *Amborella trichopoda*, dicot *Arabidopsis thaliana* and monocots *Z*. *mays*, *S*. *bicolor*, *M*. *sinensis* and *M*. × *giganteus* (Fig. 2a, b and Additional file 1: Fig. S2). It can be observed that the isozyme genes in angiosperms shared the same origin. Compared to *Amborella trichopoda*, *PAL*, *4CL*, *CCoAOMT*, and *CCR* genes in both dicots and monocots were remarkably expanded, which could be explained by a series of whole-genome duplication (WGD) and small-scale duplication events (e.g., tandem duplication). Besides, the genes in monocots were divided into several clades that are independent of the dicot clades. This result is opposite to our previous research on cellulose synthase genes, which formed six clades posterior to the divergence between dicots and monocots [30].

Here we take PAL as an example. There is only one *PAL* gene in *Amborella trichopoda* (AMTR_s00148p00088930), but in *Arabidopsis thaliana*, *Z*. *mays*, *S*. *bicolor*, and *M*. *sinensis*, the gene numbers increase to 4, 11, 8, and 13, respectively (Fig. 2b). Monocot *PAL* genes were grouped into five clades, parallel to the clade of *Arabidopsis thaliana*. In the chromosome 7 and 8 of *M*. *sinensis* genome, Misin07G42300 and Misin08G206300, Misin07G412400 and Misin08G206400 are two pairs of *PAL* genes derived from a recent genus-specific WGD event (red and blue ribbons in Fig. 2c, respectively). After that, Misin07G412400 underwent additional tandem duplication events, forming Misin07G412500 and Misin07G412600 (blue arrows in Fig. 2c).

These results suggest that the monolignol biosynthetic genes were expanded and independently evolved in monocots and dicots, implying the more complex nature of organization and regulation of the pathway than in the basal angiosperm *Amborella trichopoda*.

### Expression analysis reveals major monolignol biosynthetic genes

**Fig. 3:**
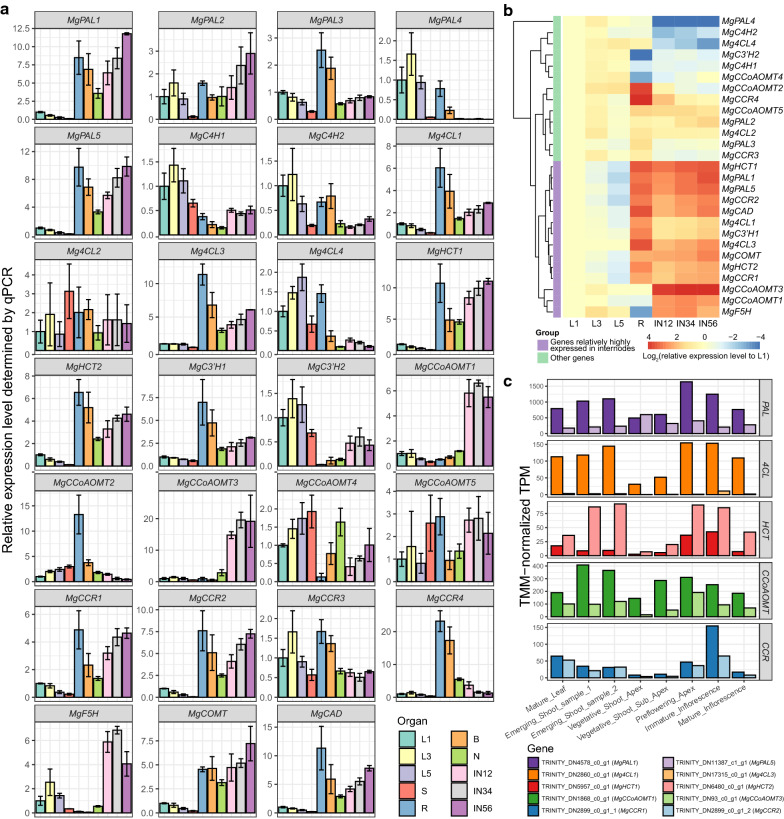
Expression analyses of monolignol biosynthetic genes in *Miscanthus* × *giganteus*. **a** The relative expression levels of each monolignol biosynthetic gene in the first (L1), third (L3), fifth (L5) fully expanded leaves, sheaths (S), roots (R), rhizome buds (B), nodes (N), and the first and second (IN12), third and fourth (IN34), fifth and sixth (IN56) internodes from the bottom of the plant. **b** The expression pattern clustering monolignol biosynthetic genes. *MgPAL1*, *MgPAL5*, *Mg4CL1*, *Mg4CL3*, *MgHCT1*, *MgHCT2*, *MgC3′H1*, *MgCCoAOMT1*, *MgCCoAOMT3*, *MgCCR1*, *MgCCR2*, *MgF5H*, *MgCOMT*, and *MgCAD* were clustered together and relatively highly expressed in internodes. **c** The quantification of *PAL*, *4CL*, *HCT*, *CCoAOMT*, and *CCR* gene pairs in various *M*. × *giganteus* organs. All the genes except for *Mg4CL3* were highly or moderately expressed in these samples

The relative expression levels of the monolignol biosynthetic genes from all monocot clades were determined in leaves, sheaths, roots, rhizome buds, nodes, and internodes (Fig. 3a). The isozyme genes exhibited similar or different expression patterns to each other. For instance, the relative expression pattern of *MgPAL1* was quite identical to *MgPAL5*, whereas distinct from *MgPAL4*. It indicates that these duplicated genes have partially specialized at the expression level.

The internode is the primary site of lignin biosynthesis. In *Arabidopsis thaliana* [31] and *Z*. *mays* [26], the major monolignol biosynthetic genes showed remarkably higher expression in internodes than in leaves (also see Additional file 2: Table S5). Additionally, most of them were highly expressed in roots, which are also rich in lignified vascular tissues. Based on this conserved expression pattern, the major monolignol biosynthetic genes in *M*. × *giganteus* could be estimated. For a more intuitive comparison, the relative expression levels of all the monolignol biosynthetic genes in leaves, roots, and internodes were clustered by gene and visualized in a heatmap (Fig. 3b). The result shows that the 14 genes, *MgPAL1*, *MgPAL5*, *Mg4CL1*, *Mg4CL3*, *MgHCT1*, *MgHCT2*, *MgC3′H1*, *MgCCoAOMT1*, *MgCCoAOMT3*, *MgCCR1*, *MgCCR2*, *MgF5H*, *MgCOMT*, and *MgCAD*, were clustered together, sharing consistent expression patterns with the major monolignol biosynthetic genes in *Arabidopsis thaliana* and *Z*. *mays*.

However, the expression levels between two genes cannot be directly compared using relative qPCR due to different amplicon lengths, amplification efficiencies, and fluorescence thresholds. Therefore, we performed transcriptome analysis in *M*. × *giganteus* to determine the absolute expression levels of isozyme genes and narrow down the number of major monolignol biosynthetic gene candidates. The overall read mapping rate and E90N50 of the transcriptome assembly were 93.58% and 1,778 bp, respectively, suggesting the high completeness and continuity of the transcripts. As we expected, the two reference genes used in this study, *eEF-1a* and *UBQ*, were steadily expressed in all vegetative organs (Additional file 1: Fig. S3). Furthermore, *Mg4CL1*, *MgHCT1*, *MgHCT2*, *MgCCoAOMT1*, *MgCCoAOMT3*, *MgCCR1*, and *MgCCR2* were highly or moderately expressed in the vegetative and reproductive organs above the ground. In contrast, *Mg4CL3* was rarely expressed in any samples, indicating that *Mg4CL3* is not likely to act as the major monolignol biosynthetic gene (Fig. 3c).

In conclusion, *MgPAL1*, *MgPAL5*, *Mg4CL1*, *Mg4CL3*, *MgHCT1*, *MgHCT2*, *MgC3′H1*, *MgCCoAOMT1*, *MgCCoAOMT3*, *MgCCR1*, *MgCCR2*, *MgF5H*, *MgCOMT*, and *MgCAD* are most likely to be the major monolignol biosynthetic genes. It is worth mentioning that neither *MgC4H1* nor *MgC4H2* was relatively highly expressed in internodes, indicating tyrosine shortcut pathway may be the primary route for 4-coumaric acid biosynthesis. Therefore, MgPAL1 or MgPAL5 should be able to utilize tyrosine as the substrate in theory. Consistently, the two orthologs of MgPAL1 and MgPAL5 in *S*. *bicolor*, SORBI_3004G220300 and SORBI_3006G148800 (Fig. 2b), were examined to have such substrate affinity [22]. The key residue, histidine in the 4-methylidene-imidazole-5-one (MIO) domain conferring the function, could also be found in MgPAL1 and MgPAL5 (Additional file 1: Fig. S4).

### Asymmetric evolution between major and non-major monolignol biosynthetic genes

**Fig. 4:**
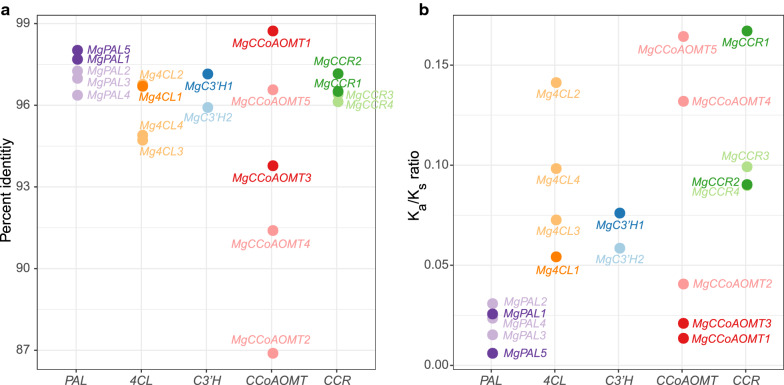
Percent identities and *K*_a_/*K*_s_ ratios of monolignol biosynthetic genes. The major monolignol biosynthetic genes (dark colors) showed **a** higher percent identities and **b** lower *K*_a_/*K*_s_ ratios than the non-major genes (light colors)

According to the neofunctionalization model and plenty of studies on duplicated genes, the genes that preserved the original functions evolve slower than other copies, which is referred to as “asymmetric evolution” [32]. We wondered whether asymmetric evolution could also be observed in the monolignol biosynthetic isozyme genes of *M*. × *giganteus*. As expected, the coding sequences of major monolignol biosynthetic genes exhibited significantly higher percent identities than the non-major genes compared to the corresponding orthologs in *S*. *bicolor* (Wilcoxon-rank sum test, *p* value = 0.01262) (Fig. 4a). Furthermore, the K_a_/K_s_ ratios showed a contrary tendency (Fig. 4b). These results demonstrate that the major monolignol biosynthetic genes have higher sequence conservation and underwent stronger purifying selection. In comparison, the rest genes evolved faster at both the transcription level and the sequence level. This finding is also consistent with the critical role of the monolignol biosynthesis in plant survival and development.

*MgCCR1* and *MgCCR2* are a pair of genes formed in the recent genus-specific WGD event. Interestingly, compared to *MgCCR2*, *MgCCR1* has rapidly accumulated mutations to the extent of *MgCCR3* and *MgCCR4* in the short-term independent evolutionary history. It suggests that the asymmetric evolution of monolignol biosynthetic genes might be accelerated at the early stage after WGD and declined in the later period. The inference agrees with the observation in the yeast WGD [33].

### Co-regulation of genes involved in monolignol biosynthesis and closely related pathways

**Fig. 5:**
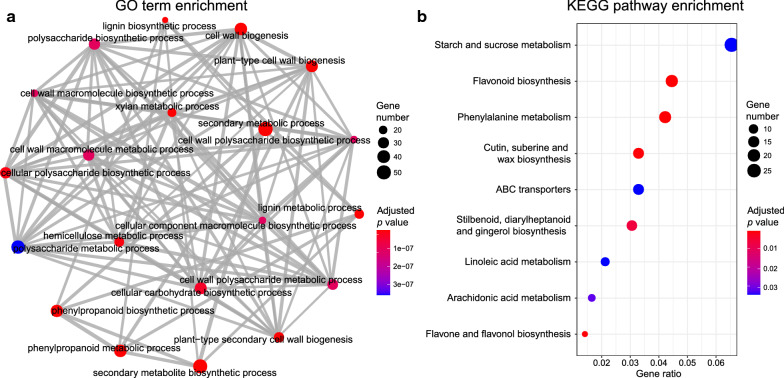
GO and KEGG functional enrichment analysis of *MgHCT1* positively correlated genes. Significantly enriched **a** GO terms and **b** KEGG pathways. The dot sizes and colors indicate the gene numbers and adjusted *p* value. The gray lines mean the two linked GO terms have common gene(s)

The transcriptome data of various *M*. × *giganteus* organs make it possible to explore the underlying gene transcription regulatory mechanisms on a border range of genes and independent samples. Firstly, we paid our attention to the functional relationship of co-expressed genes. The genes that showed significantly positively correlated expression patterns (Spearman’s correlation coefficients ≥ 0.4 and *p* value < 0.05) were regarded as the co-expressed genes. For each major monolignol biosynthetic gene, the co-expressed genes account for 3.19% to 18.42% of total expressed genes. The GO and KEGG enrichment analyses showed that these genes were significantly overrepresented in the GO terms and KEGG pathways that are related to secondary cell wall formation or share common intermediates with monolignol biosynthesis (Fisher’s exact test, Benjamini–Hochberg multiple testing corrected *p* value < 0.05) (for detailed results, see “Availability of data and materials”). For example, the co-expressed genes of *MgHCT1* were significantly enriched in GO terms of “lignin biosynthetic process”, “phenylpropanoid biosynthetic process”, “phenylpropanoid metabolic process”, “plant-type secondary cell wall biogenesis” (Fig. 5a), and KEGG pathways of “phenylalanine metabolism”, “flavonoid biosynthesis”, “flavone and flavonol biosynthesis”, “cutin suberine and wax biosynthesis” (Fig. 5b). The consistency between gene expression patterns and gene functions indicates that the major monolignol biosynthetic genes and those genes in closely related pathways are under co-regulation in *M*. × *giganteus*.

### Transcription factors of the major monolignol biosynthetic genes

TFs regulate the gene expression by specifically binding to the gene promoter regions. Based on this mechanism, the TF binding sites (TFBSs) could be predicted using the promoter sequences. To reduce false positives, we performed the expression correlation analysis between the TF genes and their target genes. The detailed results are listed in Additional file 2: Tables S6–S8.

**Fig. 6:**
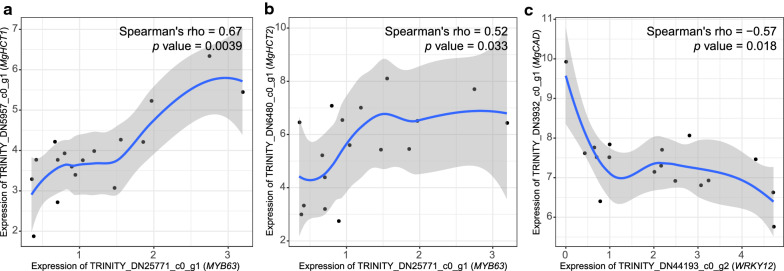
Expression correlation between monolignol biosynthetic genes and predicted transcription factor genes. The expression correlations between **a** *MYB63* and *MgHCT1*, **b** *MYB63* and *MgHCT2*, **c** *WRKY12* and *MgCAD*. The units of x- and y- axes are log2 transformed TPMs. The calculated Spearman’s rho and *p* value are shown in the plots. The blue lines and gray ribbons refer to the fitted smooth curves and 95% confidence intervals

MYB and secondary wall NAC (SWN) are two dominant TF families involved in lignin biosynthesis and other secondary cell wall formation-related pathways. By the TFBS prediction, possible MYB binding sites were significantly enriched in the promoters of major monolignol biosynthetic genes (Fisher’s exact test, *p* value = 0.0359, Additional file 1: Table S4). Furthermore, the expression levels of most major genes, including *Mg4CL1*, *MgHCT1*, *MgHCT2*, *MgC3′H1*, *MgCCoAOMT1*, *MgCCoAOMT3*, *MgCCR1*, *MgCCR2*, *MgF5H*, and *MgCOMT*, were significantly correlated with at least one corresponding *MYB* gene (Spearman’s correlation coefficients ≥ 0.4 or ≤ -0.4 and *p* value < 0.05, Additional file 2: Table S8). Among these, MYB61, MYB63, and MYB46 were predicted as the top three MYBs capable to directly activate the expression of multiple major monolignol biosynthetic genes in *M*. × *giganteus* (Additional file 2: Table S9). Similar to our result (Fig. 6a, b), the expression of the *MYB63* gene in *M*. *sinensis*, *MsSCM4*, exhibited a strong positive correlation with *MsHCT*, and the heterologous expression of *MsSCM4* in *Nicotiana benthamiana* mesophyll cells promoted lignin deposition [34].

In contrast to MYB, NAC binding sites were not significantly enriched in the promoters of major monolignol biosynthetic genes (*p* value = 0.1, Additional file 1: Table S4). Additionally, only the expression of *Mg4CL1* and *MgHCT2* showed correlation with a NAC gene (Additional file 2: Table S8). The result indicates that NACs are not likely to regulate major monolignol biosynthetic genes directly. These findings are consistent with the NAC-MYB-based gene regulatory network (NAC-MYB-GRN) model demonstrated in vascular plants. In this model, NACs function as the master switches that regulate the expression of *MYB* genes, e.g., *MYB46*/*83* [35]. Then, these MYBs promote lignin biosynthesis by activating downstream *MYB* genes like *MYB58*/*63* and *MYB103* [35]. The difference is that the MYB46 in *M*. × *giganteus* was predicted to function through directly activating monolignol biosynthetic genes in our study.

WRKY is another TF family considered to be involved in the regulation of secondary cell wall formation. The enrichment of WRKY binding sites (*p* value = 0.00132, Additional file 1: Table S4) and significant expression correlation with *Mg4CL1*, *MgCCoAOMT3*, *MgC3′H1*, and *MgCAD* were also observed in our study (Additional file 2: Table S8). In dicots and some grasses, WRKY12 represses lignin biosynthesis via SWNs [36–38]. In contrast, WRKY12 in *M*. × *giganteus* might have the capability to reduce the expression of *MgCAD* by directly binding to the promoter based on our analysis (Fig. 6c).

In recent years, ERFs were reported to activate the lignin biosynthesis in dicots [39–42]. However, few studies have revealed the function of ERFs in the secondary cell wall formation of monocots [43]. Surprisingly, we noticed that ERF binding sites were also significantly enriched in the promoters (*p* value = 2.06E-04, Additional file 1: Table S4), and some *ERF* genes were highly correlated with the expression of *MgPAL1*, *MgPAL5*, *MgCCoAOMT1*, *MgCCoAOMT3*, *MgHCT2*, *MgCOMT*, *MgF5H* and *MgCAD* (Additional file 2: Table S8). Our study indicates that ERF may also be another important TF family involved in monolignol biosynthesis in *M*. × *giganteus*. Therefore, ERFs are promising candidates for lignin content and composition manipulation by genetic engineering approaches.

## Discussion

### Integrated model of monolignol biosynthesis pathway and gene regulation in *M*. × *giganteus*

In this study, *MgPAL1*, *MgPAL5*, *Mg4CL1*, *Mg4CL3*, *MgHCT1*, *MgHCT2*, *MgC3′H1*, *MgCCoAOMT1*, *MgCCoAOMT3*, *MgCCR1*, *MgCCR2*, *MgF5H*, *MgCOMT*, and *MgCAD* were inferred to be the most probable major monolignol biosynthetic genes in *M*. × *giganteus*. The evidence from phylogenetic relationships, expression patterns, and expression levels was combined. Besides, the result was strongly supported by significant sequence conservation. Concordant results could also be observed in other monocots. Most maize monolignol biosynthetic genes have similar expression patterns to the major genes of *M*. × *giganteus* in the same clades (Additional file 2: Table S5). In recent years, CSE was reported to catalyze the reaction from caffeoyl CoA to caffeic acid; however, this enzyme is not always present in plants [23]. By aligning the switchgrass and rice *CSE* genes to the genomes of *M*. *sinensis* and *M*. *sacchariflorus*, as well as the transcriptome assembly of *M*. × *giganteus*, no *CSE* gene was found.

The involvement of isozymes is common in the monolignol biosynthesis pathway. Modification of a single gene may have little effect on the lignin content and composition. Furthermore, the presence of recent genus-specific WGD events makes things more complicated. Elucidation of the TFs that control the expression of monolignol biosynthetic genes thus becomes another important topic due to its ability to regulate multiple genes. Through TFBS prediction and expression correlation analysis, the TFs from MYB, WRKY, and ERF families were estimated to function by directly binding to the promoters of monolignol biosynthetic genes in *M*. × *giganteus* (Additional file 2: Table S9). Among these TFs, MYB61 and MYB63, which were estimated to be the dominant MYBs involved in the direct regulation of monolignol synthetic genes in *M*. × *giganteus*, were also reported in other monocots and dicots [44, 45]. Based on these results, an integrated model of the monolignol biosynthesis pathway and gene regulation in *M*. × *giganteus* was summarized (Fig. 1a, b).

However, sequence-based and expression-based approaches are the primary evidence in this work. The catalytic efficiency and substrate affinity should be further determined to figure out the actual contribution and preferred substrate(s) of each monolignol biosynthetic enzyme in *M*. × *giganteus*. The direct regulatory relationship between TFs and its target genes also needs confirmation using more straightforward evidence such as knockdown or knockout of the TF genes and ChIP-seq.

### Independent evolutionary history accounts for the functional variations of monolignol biosynthetic genes in higher plants

Although the monolignol biosynthetic genes between monocots and dicots share the same origins, their functional variations have accumulated during the approximate 160 million-year independent evolutionary history after divergence [46]. PAL is such a typical example. In dicots, PAL catalyzes the non-oxidative elimination of ammonia from phenylalanine to *p*-cinnamic acid. However, PAL from some monocots also exhibits tyrosine ammonia-lyase (TAL) activity, which directly catalyzes the reaction from tyrosine to 4-coumaric acid bypassing C4H [19–22].

Besides, the expression patterns of monolignol biosynthetic genes could also have changed even in monocots. In maize, the *C4H* gene Zm00001d009858 was relatively highly expressed in internodes (Additional file 2: Table S5), whereas in *M*. × *giganteus*, neither *MgC4H1* nor *MgC4H2* showed this trend. Based on these results, the 4-coumaric acid in *M*. × *giganteus* might be mainly synthesized from tyrosine, rather than phenylalanine. This finding implies the TF function estimated by heterologous expression should be interpreted with caution, owing to the probable differentiated regulatory mechanisms between the two species.

The WGD events in angiosperms could accelerate the evolution of monolignol biosynthetic genes. *MgCCR1* and *MgCCR2* are a pair of genes formed in the *Miscanthus*-specific WGD event. Although the expression patterns of the two genes are still similar (Fig. 3c), *MgCCR1* has rapidly accumulated variations in the short term after WGD. This observation may also be appropriate in other monolignol biosynthetic genes of *M*. × *giganteus*.

### Reference genome facilitates the gene expressional and functional studies

Although the expression levels of *MgCCR1* and *MgCCR2* were successfully distinguished using the gene-specific SNPs, the method is cloning-dependent. By taking advantage of the *M*. *sinensis* genome, this analysis could be simplified. In addition, the genome facilitated the evolutionary analysis, selective pressure analysis, and TFBS prediction of the monolignol biosynthetic genes.

However, the *M*. × *giganteus* genome is actually more complex than the *M*. *sinensis* genome. *M*. × *giganteus* is originated from the hybridization between *M*. *sinensis* and *M*. *sacchariflorus*, resulting in an allotriploid genome. That is to say, there should be approximately six similar copies in the *M*. × *giganteus* genome corresponding to a pair of alleles in *S*. *bicolor*. These genes cannot be distinguished using the *M*. *sinensis* genome only. Molecular cloning and qPCR experiment are also challenging [47]. For further gene expressional and functional studies, a high-quality and haplotype-phased genome of *M*. × *giganteus* is necessary.

## Conclusions

In this study, 14 genes were inferred as the major monolignol biosynthetic genes in *M*. × *giganteus*, including *MgPAL1*, *MgPAL5*, *Mg4CL1*, *Mg4CL3*, *MgHCT1*, *MgHCT2*, *MgC3′H1*, *MgCCoAOMT1*, *MgCCoAOMT3*, *MgCCR1*, *MgCCR2*, *MgF5H*, *MgCOMT*, and *MgCAD*. Furthermore, the TFs from MYB, WRKY, and ERF families were predicted to directly regulate the expression of these major monolignol biosynthetic genes by binding to their promoters. Based on these results, an integrated model of the monolignol biosynthesis pathway and the underlying regulatory mechanism was summarized. This study provides essential information for understanding the genetic, transcriptional, and regulatory landscape of the monolignol biosynthesis pathway in *M*. × *giganteus*. Moreover, a list of potential gene candidates was identified for genetic improvement of lignocellulosic biomass quality by manipulating the lignin content and composition.

## Methods

### Plant materials and sampling

*M*. × *giganteus* rhizomes were collected from the Miscanthus Resources Garden of Wuhan University at Ezhou, China (30°21′07′’N, 114°42′55′’E) and transplanted to a greenhouse at Wuhan University. When the plants were grown to the eight- to ten-leaf stage, various vegetative organ samples were collected for the molecular cloning and quantification of monolignol biosynthetic genes, including the first, third and fifth fully expanded leaves from the top to the bottom of the plants (namely L1, L3, and L5, respectively), sheaths (S), roots (R), rhizome buds (B), nodes (N), and the first to second, third to fourth, fifth to sixth internodes from the bottom to the top of the plants (IN12, IN34, IN56, respectively). After removal from the plants, the samples were washed and frozen in liquid nitrogen immediately for RNA extraction.

### RNA extraction and cDNA synthesis

The samples were ground to fine powders in liquid nitrogen using chilled mortars. Total RNA extraction and genomic DNA (gDNA) removal were performed with an RNAprep Pure Plant kit (DP432, TIANGEN Biotech, Beijing, China) following the manufacturer’s instruction. RNA integrity was assessed by 1.2% agarose gel electrophoresis and a NanoDrop 2000/2000c spectrophotometer (Thermo Scientific, Waltham, USA).

For molecular cloning experiments, the cDNAs were synthesized using M-MLV Reverse Transcriptase (M1701, Promega, Madison, USA). In each reaction, 10 μl of RNA and 2 μl of Oligo(dT)15 primer (C1101, Promega) were mixed, denatured at 70 °C for 5 min and cooled on ice immediately to open the secondary structure of RNA. The mixture was added with 5 μl of 5× M-MLV buffer, 2 μl of RNase-free ddH_2_O, 1 μl of M-MLV Reverse Transcriptase and then incubated on a LifePro Thermal Cycler (BIOER, Hangzhou, China) at 42 °C for 1.5 h. For quantitative PCR (qPCR) experiments, the cDNAs were synthesized using a FastQuant RT Kit (KR106, TIANGEN Biotech). To minimize gDNA contamination, we treated the cDNAs with gDNase at 42 °C for 3 min once again, then mixed it with 2 μl of 10× Fast RT Buffer, 1 μl of RT Enzyme Mix, 2 μl of FQ-RT Primer Mix, and 5 μl of RNase-free ddH_2_O. The reverse transcription reactions were performed at 42 °C for 15 min on the thermal cycler. All products were denatured at 95 °C for 3 min to inactivate the reverse transcriptase before storage at − 20 °C.

### Molecular cloning of monolignol biosynthetic genes

The cDNAs from various samples were mixed and diluted with nine volumes of ddH_2_O as the PCR template. Primers were designed based on the sequences obtained by rapid amplification of cDNA ends (RACE) or the transcriptome assembly of five *Miscanthus* species we published in the previous study [48], and orthologs in closely related species (Additional file 1: Table S1). To avoid mutations introduced by PCR, we used a high-fidelity DNA polymerase KOD-Plus-Neo (KOD-401, TOYOBO, Osaka, Japan) for amplification. For each reaction, the mixture consisted of 5 μl of 10 × PCR Buffer, 5 μl of dNTPs, 3 μl of MgSO_4_, 1.5 μl of each primer (10 μM), 2 μl of cDNA template, 2 μl of dimethylsulphoxide (DMSO), 29 μl of ddH_2_O and 1 μl of KOD-Plus-Neo in a total volume of 50 μl. PCRs were carried out on the thermal cycler using the two-step or three-step method based on the criteria described in our previous study [30]. For two-step PCR, the program was set as follows: initial denaturation at 94 °C for 2 min, 36 cycles of denaturation at 98 °C for 10 s, annealing and extension at 68 °C for 4 min. The annealing and extension of three-step PCR were modified as: 30 s at the minimum melting temperature of the primer pair and 3.5 min at 68 °C. After that, deoxyadenosine residues were added to the blunt 3′-end of the amplicons by mixing 1 μl of Taq DNA polymerase (EP0405, Thermo Scientific) to the PCR products and incubating at 72 °C for 30 min. The PCR fragments were purified from 2% agarose gel using AxyPrep DNA Gel Extraction Kit (AP-GX-250, Axygen, CA, USA). The purified fragments were ligated to pGEM-T vectors (A3600, Promega) with T4 ligase at 16 °C for 12 h and transformed into Trans5α Chemically Competent Cells (CD201-02, TransGen Biotech, Beijing, China). The positive clones that harboring the recombinant plasmids were identified by blue-white screening and colony PCR with corresponding primer pairs. The insert fragments were sequenced by Sanger sequencing.

### Phylogenetic and genome synteny analyses

The genomes and gene annotations of *Amborella trichopoda* (AMTR1.0), *Arabidopsis thaliana* (Araport11), *Z*. *mays* (NCBI B73_RefGen_v4), *S*. *bicolor* (Sorghum_bicolor_NCBIv3), and *M*. *sinensis* (v7.1 DOE-JGI, https://phytozome.jgi.doe.gov/) were downloaded for phylogenetic and genome synteny analyses. The possible monolignol biosynthetic genes in these species were identified by BLASTP (NCBI BLAST + version 2.7.1) [49]. To explore whether *CSE* genes are present in the *M*. × *giganteus*, we aligned the *CSE* genes from *Panicum virgatum* (v1.1 DOE-JGI, https://phytozome.jgi.doe.gov/) and *Oryza sativa* to the genomes of *M*. *sinensis* and *M*. *sacchariflorus* (NCBI Msac_v3) using BLASTN (NCBI BLAST + version 2.7.1). The protein sequences of each enzyme were aligned together using MAFFT (version 7.453) [50] with the method “--localpair” and the maximum iterative refinement of 1000 (--maxiterate 1000). After alignment, the phylogenetic trees were constructed by IQ-TREE (version 2.0-rc2) [51] with the parameters “-B 1000 --bnni” and illustrated using FigTree (version 1.4.3, https://tree.bio.ed.ac.uk/software/figtree/).

The python version of MCscan in the JCVI package (version 1.0.5+3.g843d2f9) [52] was utilized to intuitively visualize the duplication events of monolignol biosynthetic genes and the orthology relationships across different species. Genome synteny blocks were identified between *Amborella trichopoda* versus *Arabidopsis thaliana*, and *Amborella trichopoda* versus *S*. *bicolor* using the default parameters “--cscore = 0.7, --dist = 20, --min_size = 4”. The orthologous relationships of the monolignol biosynthetic genes between species were highlighted in the macro- and micro-synteny plots.

### qPCR experiment

Primers were designed using Oligo Primer Analysis Software (version 7.60) [53] based on the cDNA sequences of cloned monolignol biosynthetic genes. While for those genes failed to be cloned, the transcriptome assembly was used for primer design. According to our previous study, *eEF-1a* and *UBQ* were selected as the reference gene combination for inter-sample normalization [30]. The sequences of qPCR primers are listed in Additional file 1: Table S2. To ensure the reliability of qPCR experiments, we accessed the amplification efficiency and specificity of each primer pair by standard curve analysis (Additional file 1: Table S3) and 2% agarose gel electrophoresis, respectively (Additional file 1: Fig. S1). Reaction mixtures were prepared with the SuperReal PreMix Plus Kit (FP205-02, TIAGEN Biotech), containing 10 μl of 2× SuperReal PreMix Plus (with SYBR Green I), 2 μl of 50 × ROX Reference Dye for fluorescence signal normalization, 2 μl of three- to ten-fold diluted cDNA template, 0.6 μl of each primer (10 μM) and 4.8 μl of RNase-free ddH_2_O. qPCR experiments were performed on a StepOne Real-Time PCR System (Applied Biosystems, Waltham, USA) with the program: initial denaturation at 95 °C for 15 min, 40 cycles of denaturation at 95 °C for 15 s, followed with annealing and extension at 60 °C for 1 min. Additional melting curve analysis was conducted for each reaction to assess the amplification specificity (Additional file 1: Fig. S1).

Before quantification, the fluorescence thresholds of each gene across plates were adjusted to the same value manually. Then, the relative expression levels to L1 were calculated using the efficiency-corrected − ΔΔCt method [54]. The results were illustrated in bar charts with the R package ggplot2 (version 3.3.0) [55].

### Relative expression pattern clustering and transcriptome analysis

Relative expression pattern clustering and transcriptome analysis were combined to identify the major monolignol biosynthetic genes in *M*. × *giganteus*. The relative expression levels in leaves, roots, and internodes were log2 transformed and clustered using the R package pheatmap (version 1.0.12) [56] with the default parameters.

Raw RNA-seq data of *M*. × *giganteus* were collected from NCBI BioProject (PRJNA183625, 17 samples) [57] and our previous study (NCBI SRA accession number: SRR1734721, 1 sample) [48]. After quality filtering and adaptor trimming with fastp (version 0.20.0) [58], the clean reads from different samples were concatenated together and assembled using Trinity (version 2.8.6) [59]. The completeness and continuity of the assembly were assessed by the overall mapping rate using bowtie2 (version 2.3.5.1) [60] and the “contig N50 of the most highly expressed genes that represent 90% of the total normalized expression” (E90N50). To annotate corresponding genes in the assembly, we aligned the protein sequences of monolignol biosynthetic genes and the coding sequences of transcription factor genes to the longest transcript of the assembly using BLASTP and BLASTN, respectively. The best hits were then selected.

In the quantification analysis, only the 17 samples in the PRJNA183625 were selected to minimize the batch effect. The gene-level expression across these samples was determined and normalized using the Perl script “abundance_estimates_to_matrix.pl” in Trinity with the parameters “--est_method RSEM, --cross_sample_norm TMM”. *MgCCR1* and *MgCCR2* are two genes formed in the genus-specific whole-genome duplication (WGD) event. Although they were assembled into one gene in the transcriptome assembly due to high similarity, their expression could be distinguished by the sequencing depths of gene-specific SNPs. Firstly, 15 gene-specific SNPs were identified by multiple sequence alignment and written to a Variant Call Format (VCF) file. Then the sequencing depth of each SNP was counted using ASEReadCounter in GATK (version 4.1.7.0) [61]. Finally, the expression of each gene was calculated by multiplying the total expression level and the average depth proportion of gene-specific SNP. The absolute expression levels of isozyme genes and qPCR reference genes were illustrated with bar charts using ggplot2.

The possible functions of assembled genes were annotated using the eggNOG-mapper (version 2.0.1-14-gbf04860) [62, 63]. Only the genes assigned to Viridiplantae were kept for the downstream functional enrichment analyses. The expression correlation between genes was determined using Spearman’s correlation coefficient. Then Gene Ontology (GO) and Kyoto Encyclopedia of Genes and Genomes (KEGG) functional enrichment analyses were performed and visualized using clusterProfiler (version 3.10.1) [64].

### Calculation of percent identities and K_a_/K_s_ ratios

The monolignol biosynthetic gene orthologs between *M*. × *giganteus* and *S*. *bicolor* were compared to determine whether the major genes have higher sequence conservation and undergo stronger purification selection. The genes that failed to be cloned were replaced by the orthologs in *M*. *sinensis*. The protein sequences were aligned using MAFFT as described above. The results were used for guiding the codon alignments of coding sequences with PAL2NAL (version 14) [65]. The percent identities were calculated with a custom Python script and compared between the major genes and non-major genes using single-tailed Wilcoxon’s rank-sum test. The selective pressures were measured by nonsynonymous to synonymous substitution rate (K_a_/K_s_) ratios using the KaKs_Calculator (version 2.0) [66].

### Transcription factor analysis

The 500-bp upstream of each gene was regarded as the promoter. The DNA sequence of each region was extracted from the *M*. *sinensis* genome with a custom Python script. The TFBS were predicted by PlantRegMap [67] using maize transcription factors as the targets. The motifs on both positive and negative strands were taken into consideration. The enrichment of transcription factors was determined by single-tailed Fisher’s exact test.

### Statistics analysis

The Spearman’s correlation tests, Wilcoxon’s rank-sum test, and Fisher’s exact tests were performed in R (version 3.5.3) using the functions cor.test, wilcox.test, and phyper, respectively.

## Supplementary information

**Additional file 1.** Addiitonal figures and tables.

**Additional file 2.** Additional tables.

## Data Availability

The transcriptome assembly, normalized expression matrix, functional annotation results, and the custom Shell, Python, and R scripts of all the bioinformatics analyses described in this article are available at the GitHub repository: https://github.com/zengxiaofei/monolignol-biosynthesis.

## Abbreviations

PAL: Phenylalanine ammonia-lyase
TAL: Tyrosine ammonia-lyase
C4H: Cinnamic acid 4-hydroxylase
C3H: 4-Coumarate 3-hydroxylase
COMT: Caffeic acid/5-hydroxyconiferaldehyde 3/5-*O*-methyltransferase
4CL: 4-Hydroxycinnamate: CoA ligase
HCT: Hydroxycinnamoyl CoA: shikimate hydroxycinnamoyl transferase
C3*′*H: Coumaroyl shikimate 3′-hydroxylase
CCoAOMT: Caffeoyl CoA 3-O-methyltransferase
CCR: Cinnamoyl CoA reductase
CAD: Cinnamyl alcohol dehydrogenase
F5H: Ferulic acid/coniferaldehyde 5-hydroxylase
CSE: Caffeoyl shikimate esterase
WGD: Whole-genome duplication
GO: Gene Ontology
KEGG: Kyoto Encyclopedia of Genes and Genomes
TF: Transcription factor
TFBS: Transcription factor binding site
SWN: Secondary wall NAC
