# Supplementary material for: Genetic, transcriptional, and regulatory landscape of monolignol biosynthesis pathway in Miscanthus × giganteus
Source: Biotechnol Biofuels. 2020 Oct 27;13:179. doi: 10.1186/s13068-020-01819-4 (PMC7590476; doi:10.1186/s13068-020-01819-4)
Supplement: Supplementary file 1 — Additional file 1. Addiitonal figures and tables. [file 13068_2020_1819_MOESM1_ESM.docx]

**Additional file 1:**

**Genetic, transcriptional, and regulatory landscape of monolignol biosynthesis pathway in *Miscanthus* × *giganteus***

Xiaofei Zeng ^a, b^, Jiajing Sheng ^c, d^, Fenglin Zhu ^d^, Tianzi Wei ^b^, Lingling Zhao ^d^, Xiaohu Hu ^d^, Xingfei Zheng ^d^, Fasong Zhou ^d^, Zhongli Hu ^d^, Ying Diao ^a, *^, Surong Jin ^e, *^

a School of Biology and Pharmaceutical Engineering, Wuhan Polytechnic University, Wuhan 430023, PR China

b School of Medicine, Southern University of Science and Technology, Shenzhen 518055, PR China

c School of Life Sciences, Nantong University, Nantong, 226019, PR China

d State Key Laboratory of Hybrid Rice, Hubei Lotus Engineering Center, College of Life Sciences, Wuhan University, Wuhan 430072, PR China

e School of Chemistry, Chemical Engineering and Life Sciences, Wuhan University of Technology, Wuhan 430070, PR China

* Corresponding author

Tel/Fax: +86 027 6875 3611.

E-mail addresses: ydiao@whu.edu.cn (Ying Diao), jinsr@163.com (Surong Jin).

**Additional Figures**

**Fig. S1. Assessment of qPCR specificity.** a Melting curve analysis of qPCR products after 40-cycle amplification. b. 2% agarose gel electrophoresis of qPCR products. The results indicate the high specificity of the primers.

**Fig. S2. Phylogenetic analysis of monolignol biosynthetic genes in *Miscanthus* × *giganteus*.** The numbers beside the nodes are ultrafast bootstrap values. The clades were filled with different colors. The genes starting with “AMTR”, “At”, “SORBI”, “Zm”, “Misin” and “Mg” are from *Amborella* *trichopoda*, *Arabidopsis* *thaliana*, *Sorghum* *bicolor*, *Zea* *mays*, *Miscanthus* *sinensis*, and *M*. × *giganteus*, respectively.

**Fig. S3. Expression levels of the two qPCR reference genes, *eEF-1a* and *UBQ*.** The expression levels of eEF-1a and UBQ are shown as stacked bar chart. The result indicates the two genes are steadily expressed in all vegetative organs.

**Fig. S4. Sequence alignment of 4-methylidene-imidazole-5-one (MIO) domain of PALs.** The genes containing histidine residues in the MIO domain are inferred to have the tyrosine ammonia-lyase activity.

**Additional Tables**

**Table S1. PCR primers for cloning monolignol biosynthetic genes in *Miscanthus* × *giganteus*.**

| Primer name | Primer sequence |
| --- | --- |
| CCR1/2F1 | 5’-CACCGTCAGGAACCCAGATG-3’ |
| CCR1/2R1 | 5’-GCCGTAGCAGTACCAGTTCC-3’ |
| COMTF1 | 5’-TAAGGACGCGGTGCTTGACG-3’ |
| COMTR1 | 5’-TTCACTCGACGACGATCACC-3’ |
| CADF1 | 5’-ACACCTCTCCCCCTACTCCT-3’ |
| CADR1 | 5’-TCAGGATCTTCACCACAAAC-3’ |
| CCRF | 5’-ACAGACAAAGGGAAAACCTAAAAACTA-3’ |
| CCRR | 5’-GAACGAACATTAACCATTTTTATTCAC-3’ |
| COMTF | 5’-CAGCAGCACACACCAGCACTTCC-3’ |
| COMTR | 5’-ATAGTAAGTATAACATTGATTGT-3’ |
| CADF | 5’-GTCGCGCTGCTTGCTTTCTTTC-3’ |
| CADR | 5’-AACTGCAAATCCAGAATGCTGGT-3’ |
| PAL1F | 5’-ACCACAGCACAGCATAATGGCGGG-3’ |
| PAL1R | 5'-GACGGCACGATCTGGACATACACG-3' |
| PAL2F | 5’-CCCACAGCAATGGCGTGCGAGAAC-3’ |
| PAL2R | 5’-TGTTCAGCAGATGGGCAGTGGCTC-3’ |
| PAL3F | 5’-TTAGCTAGCTGTACTAATAATCCG-3’ |
| PAL3R | 5’-CTTACAAGTTAAAACCATCAAAAG-3’ |
| PAL5F | 5’-CTGCCTCCCGAGCTCTTCTTCCAC-3’ |
| PAL5R | 5’-TYAGCAGATGGGCAGGGGCTTGCC-3’ |
| C4H1F | 5’-ATGGACCTCGTGCTCCTGGAGAAG-3’ |
| C4H1R | 5’-CTAGGCCTCGAGGGGCTTGCAGAC-3’ |
| C4H2F | 5’-ATGGACCTYCTCTTCSYGGAGA-3’ |
| C4H2R | 5’-YTAGAACKYTCTTGGCTTGCAC-3’ |
| 4CL1F | 5’-GAGATGGGTTCCGTRGACRCGG-3’ |
| 4CL1R | 5’-TAACTGAAACATGGTACTCCAT-3’ |
| 4CL4F | 5'-ATGGTGTCGCCGACGGAGCCGCAG-3' |
| 4CL4R | 5'-GCATCAACACGATGATCCGTTGGA-3' |
| HCT1F | 5’-ATCGCTGGATTTGTCCAGAGTCTT-3’ |
| HCT1R | 5’-TCAAGTAACCCCACATCGAGTTTG-3’ |
| C3’H1F | 5’-CCATGGACGSCKCCSYSCTCCT-3’ |
| C3’H1R | 5’-TCACATCTCRACSGGGACCCTM-3’ |
| C3’H2F | 5’-ATGRASRCGGCCTCCTTCCTSK-3’ |
| C3’H2R | 5’-TCASAYSTCSGAMGGSACACGC-3’ |
| CCoAOMT1F | 5’-ATGGCCASCACGGCGRCSGAGS-3’ |
| CCoAOMT1R | 5’-TCACTTGACGCGGCGGCAGAGS-3’ |
| CCoAOMT3F | 5'-SCATGGCKVCCRGCGGCGRCR-3' |
| CCoAOMT3R | 5'-TCASACGASGCGGCGGCAGATG-3' |
| CCR3F | 5'-ATGGCCGTCGTCGTGTGCGTCACC-3' |
| CCR3R | 5'-GATAACCAATCAAATGAACGACTT-3' |
| CCR4F | 5'-ATGCCAACAGCAGAGRCGACGACG-3' |
| CCR4R | 5'-TCATGATTTGTGGAGTTGGTCCTG-3' |
| CCRL1F | 5'-ATGGCGGCGCCGGCGGCGAAGAGC-3' |
| CCRL1R | 5'-TCAATTCAGGATGCCAAATGCTTT-3' |
| CCRL2F | 5’-GCGGCTGCCTCAGAACTCCTCA-3’ |
| CCRL2R | 5’-TGTTTCACAGGTTGCTCGAGTC-3’ |
| CCRL3F | 5’-ATGTCGTCCAACTGCGGYGAGG-3’ |
| CCRL3R | 5’-TYACARGTACGCGCGCTGCTGY-3’ |
| F5HF | 5’-ATGGCGGCCGTKGCCAAGATCGCC-3’ |
| F5HR | 5’-AGTCCAAAGTCCAAACGCGTTCAT-3’ |

**Table S2. Primers for qPCR relative quantification of monolignol biosynthetic genes in *Miscanthus* × *giganteus*.**

| Primer name | Primer sequence |
| --- | --- |
| PAL1-RTF | 5'-CCGCTGCCCATCAACGTC-3' |
| PAL1-RTR | 5'-ACGGCACGATCTGGACAT-3' |
| PAL2-RTF | 5'-GTTCGACGCCGAGACATCCG-3' |
| PAL2-RTR | 5'-TTCTGCTTGGTATCGCTGCCGTG-3' |
| PAL3-RTF | 5'-CTCGATCAAGACAGAGCGTTT-3' |
| PAL3-RTR | 5'-TAGCTACTAGCGCAAGGCAAC-3' |
| PAL4-RTF | 5'-CCTCCTCAAGGTGTTCAGATGCG-3' |
| PAL4-RTR | 5'-CCACTGCAAACATGGGCAAT-3' |
| PAL5-RTF | 5'-CCCGGCGAAGAGTGCAAC-3' |
| PAL5-RTR | 5'-TCCCACTCCTTGAGGCAT-3' |
| C4H1-RTF | 5'-AACTTCGTCCAGGAACGCAAG-3' |
| C4H1-RTR | 5'-TGTCATGGTTGATCTCGCCTT-3' |
| C4H2-RTF | 5'-CCATCCTCGGCATCACCA-3' |
| C4H2-RTR | 5'-GCACACGATGTTGGAATGCTT-3' |
| 4CL1-RTF | 5'-CAGCTTTTCAGACGGTCACAC-3' |
| 4CL1-RTR | 5'-CAGGATTAATACGTGCCCCTT-3' |
| 4CL2-RTF | 5'-ATTAAGGATGCTGCTGTTGTCT-3' |
| 4CL2-RTR | 5'-TCTCAGAGCCTTCACTCCGTA-3' |
| 4CL3-RTF | 5'-ACAAGTCGAGCCAGAAATCGG-3' |
| 4CL3-RTR | 5'-GCCACAAATTGTTTCACGTCA-3' |
| 4CL4-RTF | 5'-CGTCCCAATGAAGGATGACT-3' |
| 4CL4-RTR | 5'-CACCACCTGTTTCGCCACGTA-3' |
| HCT1-RTF | 5'-GCCTGTCCATTGCTATCTCGT-3' |
| HCT1-RTR | 5'-TGGGTACAACATAAAGCCTCTCG-3' |
| HCT2-RTF | 5'-CCGGAAGCTCATCTACGACT-3' |
| HCT2-RTR | 5'-CCCTCCCTGAGCCTCTTGCTA-3' |
| C3’H1-RTF | 5'-TCACGTTCATGGGCACACCAC-3' |
| C3’H1-RTR | 5'-TCACATCTCAACGGGGACCCT-3' |
| C3’H2-RTF | 5'-CATTTCGTCGATGCGCTCT-3' |
| C3’H2-RTR | 5'-CCCACTCCACCGAGATCACC-3' |
| CCoAOMT1-RTF | 5'-AAGAGCGACGACCTCTACCAG-3' |
| CCoAOMT1-RTR | 5'-AGGTCGTCATCAGGTTCCAT-3' |
| CCoAOMT2-RTF | 5'-TCCTTGCATGTTCCGGTCCAC-3' |
| CCoAOMT2-RTR | 5'-GCACACAGGAGCTAGTAGCAT-3' |
| CCoAOMT3-RTF | 5'-ATGCTGCTCAAGCTCACCG-3' |
| CCoAOMT3-RTR | 5'-CTCCCGGCTCACGTCGAAC-3' |
| CCoAOMT4-RTF | 5'-GTGAAGAACATCCACGGCAAA-3' |
| CCoAOMT4-RTR | 5'-ATCGTGTTGAGCATGTACTCGT-3' |
| CCoAOMT5-RTF | 5'-CCTCCCCGCAACAACATCG-3' |
| CCoAOMT5-RTR | 5'-CAGCCGTCTTGTTGCCTTC-3' |
| CCR1-RTF | 5'-ACGGAGGCCGACGACAA-3' |
| CCR1-RTR | 5'-ATCTCCCTCTCACGCACGGATG-3' |
| CCR2-RTF | 5'-GCAGGGAGGAATCGCAAT-3' |
| CCR2-RTR | 5'-ATCATCAGCCAGCGAACAAAC-3' |
| CCR3-RTF | 5'-AGCCTCTGAAGGACCTAGGAA-3' |
| CCR3-RTR | 5'-CTGCGTCGTTCAGGCACCTTG-3' |
| CCR4-RTF | 5'-AACTTTGCCGCATCCTCGTT-3' |
| CCR4-RTR | 5'-CAGCACCGGCACAAAGTCCA-3' |
| F5H-RTF | 5'-TGATGCACAGCCCCGACGACCTC-3' |
| F5H-RTR | 5'-CCAGGTCCGACTCGTTCACGTT-3' |
| COMT-RTF | 5'-GCCACCTACATCTACGCCAAC-3' |
| COMT-RTR | 5'-CATCTCACGCATCGCAGCTC-3' |
| CAD-RTF | 5'-CTTCCTTTAGTAGTTGTGGGCTTG-3' |
| CAD-RTR | 5'-AACAGAGCATCCATCGACTCA-3' |

**Table S3. Amplification efficiencies of qPCR primer pairs.**

| Gene name | Dilution ratio | Amplification efficiency | R^2^ | Amplicon length (bp) |
| --- | --- | --- | --- | --- |
| *MgPAL1* | 1:5 | 97.07 | 0.996 | 127 |
| *MgPAL2* | 1:10 | 95.427 | 0.998 | 131 |
| *MgPAL3* | 1:5 | 86.797 | 1 | 96 |
| *MgPAL4* | 1:5 | 90.061 | 0.996 | 113 |
| *MgPAL5* | 1:5 | 89.185 | 1 | 86 |
| *MgC4H1* | 1:5 | 90.461 | 0.997 | 106 |
| *MgC4H2* | 1:5 | 93.23 | 0.996 | 137 |
| *Mg4CL1* | 1:5 | 89.312 | 0.999 | 95 |
| *Mg4CL2* | 1:5 | 90.122 | 0.997 | 88 |
| *Mg4CL3* | 1:5 | 91.83 | 0.997 | 96 |
| *Mg4CL4* | 1:5 | 90.017 | 0.994 | 115 |
| *MgHCT1* | 1:5 | 87.593 | 0.999 | 136 |
| *MgHCT2* | 1:5 | 93.573 | 0.999 | 115 |
| *MgC3’H1* | 1:5 | 90.129 | 0.999 | 89 |
| *MgC3’H2* | 1:4 | 88.39 | 0.972 | 124 |
| *MgCCoAOMT1* | 1:5 | 96.479 | 0.999 | 118 |
| *MgCCoAOMT2* | 1:3 | 91.714 | 0.992 | 90 |
| *MgCCoAOMT3* | 1:5 | 89.051 | 0.993 | 129 |
| *MgCCoAOMT4* | 1:3 | 96.335 | 0.983 | 80 |
| *MgCCoAOMT5* | 1:5 | 91.342 | 0.978 | 86 |
| *MgCCR1* | 1:5 | 84.229 | 0.999 | 84 |
| *MgCCR2* | 1:5 | 83.943 | 0.998 | 132 |
| *MgCCR3* | 1:5 | 97.644 | 0.994 | 127 |
| *MgCCR4* | 1:5 | 88.7 | 0.996 | 143 |
| *MgF5H* | 1:4 | 89.058 | 0.997 | 90 |
| *MgCOMT* | 1:5 | 93.908 | 0.999 | 95 |
| *MgCAD* | 1:3 | 98.577 | 0.986 | 80 |

**Table S4. Enrichment analysis of predicted transcription factors.**

| TF family | Unique motifs assigned in the TF family | Members in the TF family of *Z*. *mays* | Other TFs in *Z*. *mays* | All unique motifs predicted by PlantRegMap | *p* value |
| --- | --- | --- | --- | --- | --- |
| ERF | 26 | 204 | 3104 | 214 | **2.06E-4** |
| WRKY | 20 | 161 | 3147 | 214 | **0.00132** |
| MYB | 19 | 203 | 3105 | 214 | **0.0359** |
| NAC | 16 | 189 | 3119 | 214 | 0.100 |
